# Breastfeeding and Sleeping Patterns Among 6–12-Month-Old Infants in Norway

**DOI:** 10.1007/s10995-023-03805-2

**Published:** 2023-11-19

**Authors:** Ahmed A. Madar, Astrid Kurniasari, Niki Marjerrison, Ibrahimu Mdala

**Affiliations:** Department of Community Medicine and Global Health, Institute of Health and Society, University of Oslo, Oslo, Norway

**Keywords:** Breastfeeding, Sleep, Sleep patterns, Night waking, Infant, Later infancy, Norway

## Abstract

**Background:**

Parental behavior and infant sleep patterns can vary widely both within and between cultures and settings. Breastfeeding during the second half-year of infancy has been associated with frequent night waking, which is perceived as sleep problem among the Western societies. An understanding of sleeping patterns among breastfed infants during the second half-year of infancy is important in supporting continued breastfeeding.

**Objectives:**

The study aimed to investigate the sleeping patterns among breastfed infants during second half-year of infancy.

**Methods:**

This is a cross-sectional study. Three hundred and forty-two mothers of 6–12 months old breastfed infants completed the questionnaires on socio-demographic factors, breastfeeding practices, and infant sleeping patterns, which were assessed by using the Brief Infant Sleep Questionnaire (BISQ). The Cox regression model was used to assess the factors that were associated with night sleep duration whereas demographic factors and breastfeeding practices that were associated with night waking frequency were investigated using the Poisson regression model.

**Results:**

On average, the breastfed infants slept for 11 h during the night and most infants were reported to have night waking (96.8%) and were breastfed at least once at night (93.5%). In the adjusted analyses, infants in the age group 9–12 months were less likely to sleep longer compared to infants in the 6–8 months age group [HR 1.52 95% CI (1.17, 1.98)]. A one-hour increase in daytime sleep and in night wakefulness increased the likelihood of waking up at night by 19% and 24%, respectively. Infants who had been vaccinated within the last 7 days and infants who were breastfed to sleep were more likely to have a shorter nighttime sleep duration. Nighttime breastfeeding frequency was significantly associated with a 17% increase in the likelihood of night waking [IRR 1.17 95% CI (1.13, 1.22)]. Infants who slept on their parents’ bed were 1.28 times more likely to wake up at night compared to infants who slept in a separate room [IRR 1.28 95% CI (1.05, 1.59)]. Infants of parents who reported that their infants’ sleep was not a problem were 34% less likely to wake up compared to infants of parents who reported that their infants’ sleep was a problem [IRR 0.66 95% CI (0.49, 0.87)].

**Conclusions for Practice:**

Frequent night waking, bed sharing and night breastfeeding were common among 6–12 months old breastfed infants. Frequent night breastfeeding may lengthen an infant’s nighttime sleep duration. The study findings indicate that adequate information and support should be given to breastfeeding mothers in relation to the sleeping pattern of breastfed infants in order to promote continued breastfeeding practices.

**Supplementary Information:**

The online version contains supplementary material available at 10.1007/s10995-023-03805-2.

## Significance

Breastfeeding during the second half-year of infancy has numerous benefits for both mother and infant. Frequent night waking is known as a common phenomenon among 6–12 months old breastfed infants. However, frequent night waking among breastfed infants during this age is commonly perceived as an infant having sleep problems. This study is the first to understand parental behavior and infant sleep patterns in different cultures and settings in Norway. 6–12 months old breastfed infants still frequently waking and being breastfed during the night. Our findings add to the limited body of evidence on breastfeeding and frequent infant night waking.

**Supplementary Information:**

The online version contains supplementary material available at 10.1007/s10995-023-03805-2.

## Introduction

Sleep patterns among infants is undergoing intense study worldwide, particularly in Western cultures, where the norm is to establish a pattern of *sleeping through the night* in early infancy (Blunden et al., 2011). Despite the fact that normal brief arousals occur as the result of ultradian NREM-REM cyclical sleep patterns (Anders, 1994), frequent night waking after the age of 6 months is perceived as problematic sleep (Thunstörm, 1999).

The prevalence of parental-reported child sleep problems is between 10 and 46% across infants aged 6–24 months in several Western countries (Byars, 2012; Hiscock & Wake, 2001; Thunstörm, 1999; Zuckerman et al., 1987). While several different criteria in defining sleep problems were used across these studies, frequent night waking and longer time spent to fall asleep were consistently stated as sleep problems on parental reports within these ages in the studies. A cycle of sleep is lasting about 90 min and composed of two sleep stages, REM sleep (rapid eye movement sleep) and NREM sleep (non-rapid eye movement sleep).

Brief arousals, also known as night waking, is a normal phenomenon which is common during infancy (Galland et al., 2011; Hysing et al., 2014; Sadeh et al., 2009). Brief arousals normally happen between 4 and 6 times each night, at the end of every sleep cycle (A cycle of sleep is lasting about 90 min and composed of two sleep stages), as the result of ultradian NREM-REM cyclical sleep patterns (Anders, 1994; Mindell & Owens, 2010). While these brief arousals are common, they become problematic when the expectation of going back to sleep without assistance after each arousal is not achieved.

Previous studies have suggested that breastfeeding is one of the factors associated with increased night waking (Galbally et al., 2013; Hysing et al., 2014; Mindell et al., 2012). Further, infant sleep problems were also linked to subsequent negative consequences of infants’ sleep pattern in later life, mothers’ well-being (Bayer et al., 2007; Hiscock & Wake, 2001), and feeding, emotional, and behavioral problems (Hysing et al., 2014; Thunstörm, 1999; Zuckerman et al., 1987).

Following the findings of negative consequences related to infant sleep problems (waking difficulties initiating sleep and maintaining sleep), numerous intervention studies have been conducted. Intervention studies have shown improvement of infant sleep problems, as well as maternal well-being and perception of infant sleep problems (Gradisar et al., 2016; Hall, 2015; Mindell et al., 2009; Symon & Crichton, 2017). The findings of intervention studies’ improvements on infant sleep problems may be effective in reducing frequent night waking (Middlemiss et al., 2015; Schnatschmidt et al., 2022). Also, recent systematic review of prevention and treatment of infant behavioural sleep problems included controlled trials and meta-analyses conclude that behavioural interventions such extinction and bedtime fading and massages by mothers seem to be a promising strategy for treatment of sleep problems in infants above 6 months of age (Reuter et al., 2022). However, none of these studies did no examined a possible role of breastfeeding therefore, there is a lack of information for the importance of continued breastfeeding during the second half year of infancy.

There are large intercultural differences in infants’ nighttime sleeping places. However, in Scandinavian bed-sharing is common and in Norway, more than 60% of infants below one year of age reported to routinely share a bed with their parents at night (Osberg et al., 2021).

Further, parental behavior and infant sleep patterns can vary widely both within and between cultures and settings (Mindell, 2010), and few studies on this topic have been conducted in Norway. The Norwegian health authorities recommend that infants are exclusively breastfed for 6 months and partial breastfeeding until at least 12 months of age (The Norwegian Directorate of Health), and almost all mothers in Norway initiate breast-feeding (98%), 80% still breast-feeding at 6 months, but only 9% do so exclusively. Therefore, the main purpose of this study is to explore sleep patterns among breastfed infants aged 6–12 months old and the association with night breastfeeding practices, in Norway.

## Methods

This was a cross-sectional study conducted among mothers who breastfed their 6–12 month old infants. Inclusion criteria was healthy breastfeeding mothers with healthy infants at the time of the study who were giving birth at 37 weeks of pregnancy and above, with normal birth weight (≥ 2500 g) and who reside in Norway. Eligible mothers were recruited from six community health services in Akershus district, Norway, by distributing paper-based self-administered questionnaires, and from five Norwegian online parenting groups on social media platforms, by distributing online registration questionnaires (operated by the University Information Technology Center (USIT)), University of Oslo), between November 2016 and January 2017.

The questionnaire was available in both Norwegian and English, and was structured into four sections: socio-demographic factors (e.g. country of origin, age, education, occupation, housing), usual breastfeeding and feeding practices (e.g. breast milk only or mixed feeding, usual breastfeeding frequencies, and night breastfeeding), usual infant sleeping patterns (e.g. sleep durations, night waking, sleep latency, night wakefulness, sleep ecology, and parental report), and breastfeeding information and support. Infant sleeping patterns were assessed using the Brief Infant Sleep Questionnaire (BISQ), a validated and reliable infant sleep screening tool (Gradisar et al., 2016; Sadeh, 2004). BISQ consists several sleep items; including children sleep schedule, location, and sleep position (back, side, stomach), role of parents or care givers, partner/spouse support and about information/advice support of breastfeeding. This tool covers both for clinical and research purposes and it designed and tested for child from zero until three years of age.

Socio-demographic factors and breastfeeding and feeding practice questions were adopted and modified from The Norwegian Mother and Child Cohort Study (Häggkvist, 2006) and vitamin D-2 drops research-questionnaires (Madar et al., 2017).

### Sample Size Calculation

Sample size was calculated1 based on the prevalence of 46% (45) breastfed infants at 12 months of age in Norway. With a confidence level of 95%, and margin of error 6%, the sample needed was 265 respondents (specifically, mothers of 6–12-month-old breastfed infants).

### Statistical Analyses

Continuous variables that were normally distributed were described using means and standard deviations, while those that were highly skewed were described using medians and 25th and 75th percentiles. Categorical variables were described by frequencies (percentages).

Nighttime sleep duration, defined the moment the baby first falls asleep until the time being fully awake, while night waking, defined as the number of times the infant wakes up at night, were the main outcomes of interest. Infants’ age variable was later divided into two age groups of “6–8 months” and “9–12 months. We used Kaplan–Meier analysis and the log-rank test to compare differences in duration of nighttime sleep between infants aged 6–8 months, and infants aged 9–12 months. The Cox proportional hazards model was fitted to the data in order to identify factors that were associated with nighttime sleep duration and night waking. To investigate the influence of socio-demographic factors and nighttime breastfeeding practices on counts of night waking, the Poisson regression model was fitted to these data. Estimates of incidence rate ratios (IRR), representing the change of night waking counts in one group relative to a reference group, were obtained from the model. In both cases, two steps preceded the modeling of time-to-event and count data. First, univariate (unadjusted models) were fitted to the data. Secondly, independent variables that were significant (*P* < 0.05) in the univariate analyses together with clinically relevant variables were used to fit the adjusted models. Analyses of the data were performed using the IBM SPSS statistical software (V.22 SPSS Inc, Chicago, Illinois, USA) and StataSE 15. The significance level was set at 5%.

### Ethical Clearance

The study was approved by the Norwegian Centre for Research Data (reference 49892). Prior to the project, consent letters were obtained from all participants and permissions were obtained from the Health Center leaders and Facebook groups’ administrators. The project was registered and analyzed in Services for Sensitive Data (TSD), which is a secure project area at University of Oslo.

## Results

342 mothers out of 508 participated in this study, representing a response rate of 67.3%. The basic characteristics of the mothers and their infants are summarized in Table 1. The mothers were between 18 and 48 years of age, with a mean age of 30.6 ± 4.4 years. The majority (83%) of the mothers were native Norwegians, 80.1% had attained university/college degrees, and 82.5% owned properties. The mean age of infants was 8.1 months (range 6–12 months).

**Table 1:** Characteristics of the mother-infant pairs, N = 342 pairs

	N (%)
Mothers	
Country of origin	
Norway	284 (83.0)
Other countries	58 (17.0)
Mother’s age	
18–34 years old	283 (82.7)
35–48 years old	59 (17.3)
Civil status	
Married	174 (50.9)
Cohabitant	159 (46.5)
Education	
Primary, Secondary school	68 (19.9)
University/College	274 (80.1)
Employment^a^	
Full time/part time/paid parental leave/sick leave/in rehabilitation/earn disability benefit	292 (85.4)
Not employed/student	49 (14.3)
Mother’s occupation^#^	
Skill level 1–2^b^	54 (15.8)
Skill level 3–4^b^	216 (63.2)
Student/unemployed^c^	49 (14.3)
Housing	
Owned house/apartment	282 (82.5)
Rented house/apartment	48 (14.0)
Others (parent’s house or family house)	12 (3.5)
Infants	
Gender^a^	
Girl	156 (45.6)
Boy	185 (54.1)
Age^a^	
6–8 months	214 (62.6)
9–12 months	127 (37.1)
Birth week^d^	
37–40 weeks	203 (59.4)
41–43 weeks	136 (39.8)
Birth order^a^	
First born (only child)	187 (54.7)
Last born	154 (45.0)
Breastfeeding practices in addition to solid food	
Breast milk only	303 (88.6)
Breast milk and breast milk substitution	39 (11.4)

^a^One mother did not state her employment status nor the gender, age or birth order of her infant

^b^Skill level classifications were based on International Standard Classification of Occupations

^c^23 (6.7) mothers did not state their occupation

^d^The gestation period of 3 (0.9) infants not given

Table 2 shows the distribution of the median sleep pattern duration between infants in the age groups 6–8 months and 9–12 months. The median daytime sleep duration was 0.5 h longer in the 6–8 months age group, while the median nighttime sleep duration across the age groups were similar (11 h). Thus, the resulting median of total sleep duration was also longer by 0.5 h among infants in the 6–8-month-old age group compared to the 9–12-month-old age group.

**Table 2:** The median sleep pattern duration among infants aged 6–8 months and 9–12 months, in hours

Sleep pattern variables	N = 342	6–8 months	9–12 months
		Median (IQR)	Median (IQR)
Daytime sleep duration	341	3.0 (2.0, 4.0)	2.0 (2.5, 3.0)*
Nighttime sleep duration	341	11.0 (10.0, 12.0)	11.0 (10.0, 11.5)
Total sleep duration	341	14.0 (13.5, 15.0)	13.5 (12.5, 14.0)
Sleep latency	336	0.5 (0.25, 0.75)	0.5 (0.0, 3.0)
Night wakefulness	331	0.5 (0.0, 4.0)	0.5 (0.0, 6.0)

IQR is defined as Q1 (lower quartile) and Q3 (upper quartile)

*P value < 0.05

### Sleep Ecology and Parental Reports on Infant Sleep Patterns

Steps taken to assist infants to sleep, also known as sleep assists, were investigated. Most of the breastfed infants were put to sleep while breastfeeding (47.2%), and 35.1% were put to sleep while being either bottle fed, rocked, held or cuddled, or with the presence of a parent(s) in the room. Only 17.1% of the infants went to sleep without assistance from the parent(s).

In relation to sleep position (how they were placed or how they typically ended up) a total of 43.5% infants slept on their back (supine), while 39.7% slept on their side, and 16,8% slept on their stomach (prone). The sleep location was also investigated, with 52.2% of infants sharing a bed with their parent(s), 30.2% room sharing with their parent(s), and only 17.6% of the infants sleeping alone in their own room.

Parental perception of problematic sleep among their infants was investigated. Around 50.4% four percent of mothers perceived their infants’ sleep patterns as non-problematic, 45.5% perceived it as a minor problem, and only 4.1% perceived their infants’ sleep patterns as a serious problem.

### Night Waking and Night Breastfeeding

**Fig. 1:**
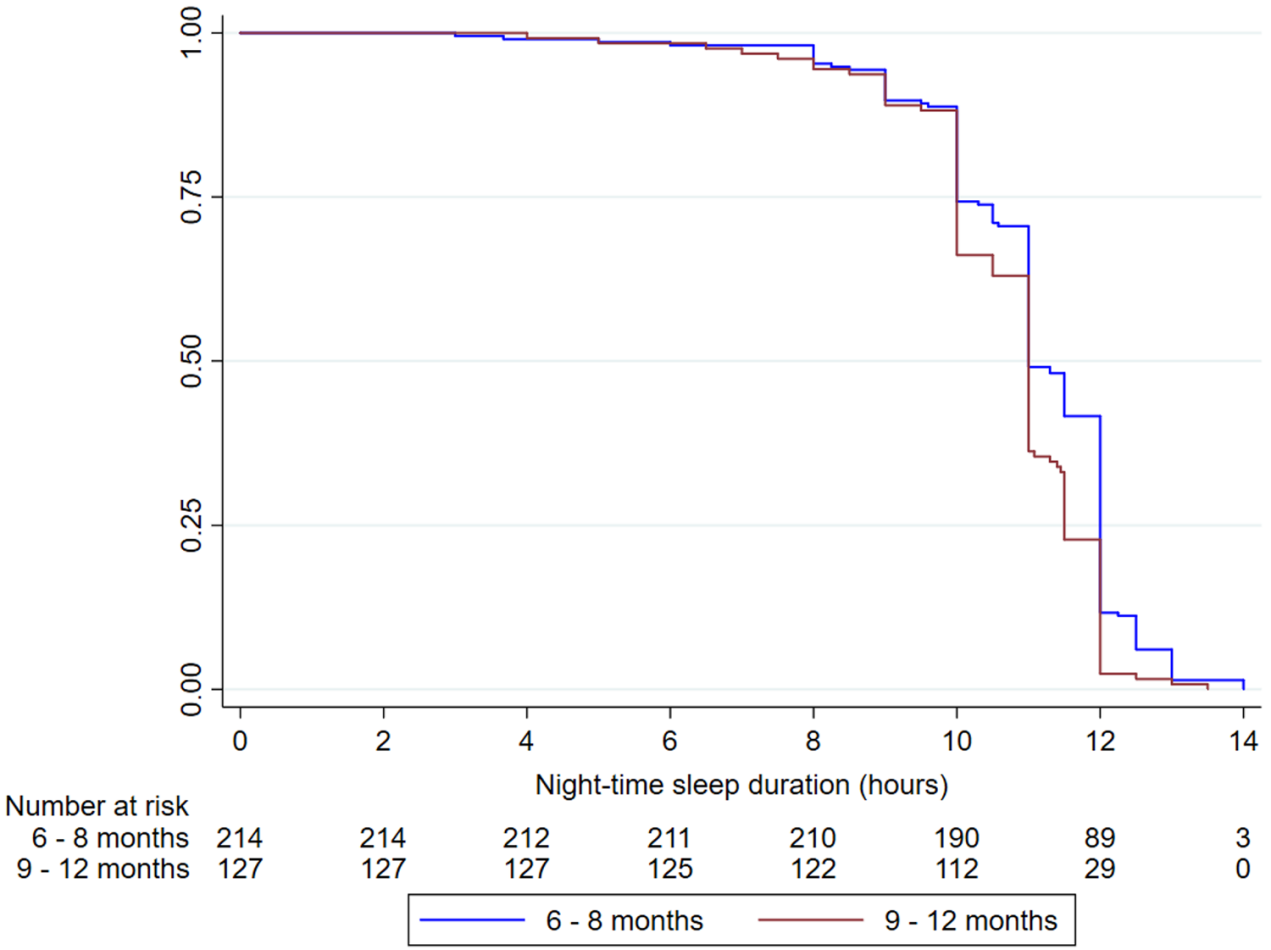
Kaplan–Meier plot with a number-at-risk table showing the nighttime sleep duration of infants in the two age groups. A drop in the curve indicates the time infants woke up (that is, the end of the nighttime sleep duration)

The median frequency of night waking among 6–8 month old infants was three (range 0–15), while the median frequency among 9–12 month old infants was four (0–15) episodes. The median of night breastfeeding frequency among the 6–8 month old infants was three (range 0–15), and the median among 9–12 month old infants was also three (range 0–8). In Fig. 1, infants who frequently woke up at night were more likely to be breastfed. Almost all breastfed infants were experiencing night waking (96.8%) and were breastfed (93.5%) at least once during the night. Few infants were breastfed but did not wake up during the night (3.2%), and 5% were not breastfed during the night (Figure S1).

### Nighttime Sleep Duration

**Fig. 2:**
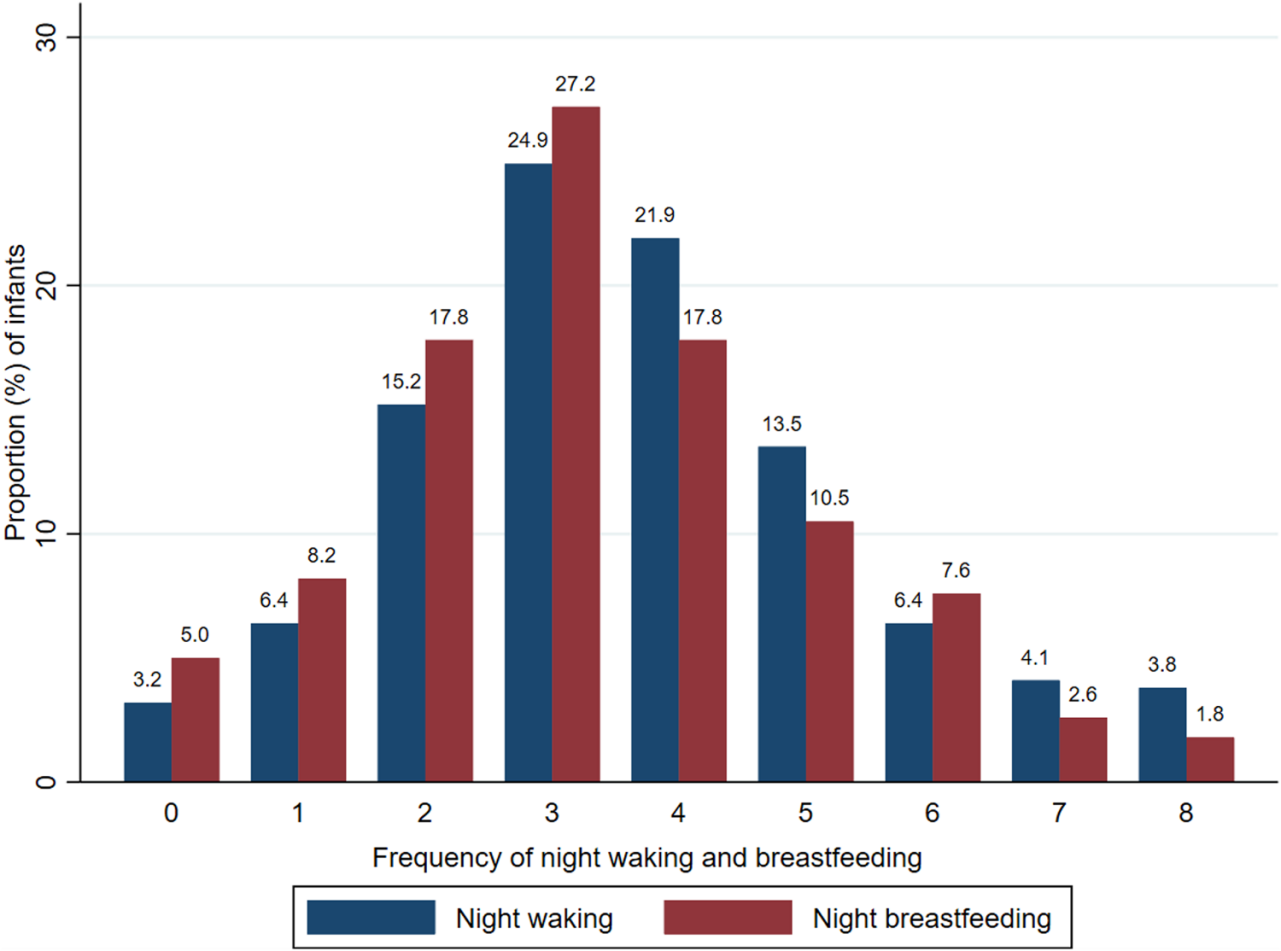
Frequency of infants experiencing night waking and night breastfeeding

A visual inspection of the Kaplan–Meier plot (Fig. 2) indicates that infants in the age group 6–8 months had a slightly longer nighttime sleep duration than infants in the age group 9–12 months. It took approximately 14 h for the last baby to wake up in the age group 6–8 months and approximately 13.5 h in the age group 9–12 months. However, the median nighttime sleep duration in both age groups was found to be 11 h, which indicates that 50% of the infants slept longer than 11 h in both age groups. Based on the log-rank test, the analysis showed that the overall survival distributions for the two age groups were statistically significant (*P* = 0.04). Results for sleep duration for each month between 6 and 12 months are presented in Figure S2.

### Factors Associated with Nighttime Sleep Duration

Table 3 is a full analysis of covariance using the semi-parametric Cox proportional hazards model. In the univariate analysis, infant’s age, vaccination within the last 7 days, sleep associations, daytime sleep duration, sleep latency, night wakefulness, and parental report on infant sleep, were all significantly associated with nighttime sleep duration (*P* < 0.05).

**Table 3:** HR estimates and their 95% CIs showing demographic factors and breastfeeding practices that were associated with nighttime sleep duration

Factors	N = 342	Unadjusted		Adjusted	
		HR (95% CI)	*P* value	HR (95% CI)	*P *value
Infant age (ref: 6–8 months)	214				
9–12 months	147	1.38 (1.11, 1.73)	< 0.01	–	< 0.01
Infants’ vaccination the last 7 days (ref: Yes)	10				
No	331	0.50 (0.27, 0.95)	0.03	0.45 (0.22, 0.90)	0.03
Nighttime breastfeeding frequency	337	0.99 (0.93, 1.06)	0.88	0.91 (0.83, 0.99)	0.03
Sleep associations (ref: While breastfeeding)	160				
In bed alone	60	0.71 (0.53, 0.96)	0.03	0.62 (0.41, 0.94)	0.02
Daytime sleep duration	341	1.14 (1.03, 1.25)	0.01	1.19 (1.07, 1.32)	< 0.01
Sleep latency	336	1.30 (1.05, 1.60)	0.01	1.22 (0.95, 1.57)	0.12
Night wakefulness	331	1.21 (1.09, 1.35)	< 0.01	1.24 (1.08, 1.41)	< 0.01
Parent reported infant sleep as a problem (ref: Very serious problem)	14				
Not a problem	172	0.49 (0.29, 0.86)	0.01	0.54 (0.27, 1.06)	0.08

In the adjusted analysis, the results showed that the 9–12 month-old infants were 52% more likely to wake up from their nighttime sleep compared to the 6–8 month-old infants. We also observed that 1-h increase in daytime sleep and in night wakefulness increases the likelihood of waking up at night by 19% and 24%, respectively. In other words, infants who slept longer during the daytime were more likely to sleep less during the night. Vaccination and being breastfed to sleep were also significantly associated with nighttime sleep duration. Infants vaccinated within the last 7 days or who were breastfed to sleep were more likely to have a shorter nighttime sleep duration. Each increase of night breastfeeding frequency was associated with a 9% decrease in infants’ probability to wake up from their nighttime sleep. This implies that infants who were more frequently breastfed at night slept longer than those who were less frequently breastfed.

No further associations were observed between nighttime sleep duration and other breastfeeding variables, such as breastfeeding status in addition to solid food (breast milk only or breast milk and formula), daytime breastfeeding frequency, being breastfed at nighttime or not, and total breastfeeding frequency per day.

### Factors Associated with Night Waking

In the univariate analyses, significant associations were observed between night waking and the following factors: civil status, breastfeeding status, daytime and nighttime breastfeeding frequency, night breastfeeding (yes/no), sleep location, sleep association, sleep latency, night wakefulness, parental report on infant sleep, breastfeeding information during pregnancy, and joining a breastfeeding support group. After adjusting for the factors presented in Table 4, night breastfeeding frequency and bed sharing were the only two factors that were significantly associated with night waking. The frequency of night waking significantly increased by 17% each time the infant was breastfed during the night and by 31% if the mother-infant pair shared a bed. There was no night awaken difference between mothers who had a previous child whom they breastfed (or didn’t breastfeed) compared to those for whom this was their first child (p = 0.19).

**Table 4:** Incidence rate ratio (IRR) estimates and their 95% confidence intervals (95% CI) showing demographic factors and breastfeeding practices that are associated with night waking frequency

Factors	N = 342	Unadjusted		Adjusted	
		IRR (95% CI)	*P* value	IRR (95% CI)	*P *value
Civil status (ref: Married)	174				
Cohabitant	159	1.13 (1.01, 1.26)	0.04	1.04 (0.92, 1.17)	0.54
Feeding status (ref: Breast milk only)	303				
Breast milk and substitutes	39	0.81 (0.67, 0.98)	0.03	0.85 (0.69, 1.05)	0.14
Daytime breastfeeding frequency	342	1.04 (1.01, 1.07)	0.01	1.00 (0.96, 1.03)	0.82
Nighttime breastfeeding frequency	337	1.19 (1.15, 1.23)	< 0.01	1.17 (1.13, 1.22)	< 0.01
Sleep location (ref: Crib in a separate room)	60				
Infant’s crib in parent’s room	104	1.29 (1.07, 1.56)	0.01	1.18 (0.97, 1.45)	0.11
In parents’ bed	178	1.60 (1.34, 1.90)	< 0.01	1.28 (1.05, 1.59)	0.02
Sleep associations (ref: While breastfeeding)	160				
In bed alone	60	0.74 (0.62, 0.87)	< 0.01	1.16 (0.95, 1.41)	0.15
Sleep latency	336	1.22 (1.10, 1.34)	< 0.01	0.98 (0.87, 1.10)	0.75
Night wakefulness	331	1.14 (1.08, 1.22)	< 0.01	1.00 (0.99, 1.01)	0.14
Infant’s sleep reported as a problem (ref: Very serious)					
Small problem	155	0.78 (0.61, 0.90)	0.04	0.88 (0.62, 1.06)	0.12
Not a problem	172	0.59 (0.46, 0.75)	< 0.01	0.66 (0.49, 0.87)	0.04
Breastfeeding information/advice before birth (ref: Yes)	280				
No	61	1.18 (1.03, 1.35)	0.02	1.06 (0.92, 1.23)	0.39
Mother joins breastfeeding support group (ref: Yes)	221				
No	121	0.80 (0.71, 0.90)	< 0.01	0.86 (0.92, 1.23)	0.39
Infants’ vaccination the last 7 days (ref: Yes)	10				
Yes	331	1.34 (0.91, 1.97)	0.14	1.31 (0.88, 1.96)	0.19

## Discussion

Continued breastfeeding of infancy is recommended until 2 years or beyond, considering the importance and the benefits for infants and mothers. However, the link between breastfeeding and frequent night waking, which is perceived as an infant sleep problem, could challenge the practice of night breastfeeding. Therefore, this study investigated the sleeping patterns among breastfed infants in the second half-year of infancy.

Indeed, our findings suggested that both night waking and night breastfeeding were common among 6–12 months old infants. Only a few of breastfeeding infants in our study were not breastfed during the night. Nearly all of the breastfeeding infants in the 6–12-month age group were waking and being breastfed at least once during the night, and around half of the infants were having night waking and night breastfeeding between 3 and 4 times each night.

Furthermore, the relationship of breastfeeding and sleep in the adjusted analysis suggested that infant were breastfed during the night because the infants woke up. This result confirmed earlier findings of breastfeeding association with infant sleeping patterns, which showed that breastfed infants were likely to wake more frequently during the night (Galbally et al., 2013; Sadeh et al., 2009; Touchette et al., 2005).

Human breast milk’s easily digestible composition and its relation to infants being woken up at night to meet their satiety needs has also previously been discussed in an aforementioned study (Galbally et al., 2013). This may be common during the first half-year of infancy, when the major source of nutrition for the infant can be mainly human breast milk. However, during the continued breastfeeding period when infants had begun to complementary food in addition to breast milk, as they were in our study, the possibility of hunger due to breast milk composition is unlikely. Based on our finding that frequently night-breastfed infants were likely to wake more during the night, it is suggested that there are other factors such as biological and parental behavior that may also influence sleeping patterns.

The factor of parental behavior was discussed in previous studies by Sadeh et al. (2009) and Ramamurthy et al. (2012). They suggested that breastfeeding status itself did not increase the likelihood of night waking among infants, but that breastfeeding as a parental response in assisting their infants to sleep did (Ramamurthy et al., 2012; Sadeh et al., 2009). This may also explain our findings.

The relationship between frequent night breastfeeding and night waking may also be related to infants’ and mothers’ bodily response to breast milk. Breast milk production is known to be positively associated with infant's frequent demand for milk (Kent et al., 1999). Thus, the association between night breastfeeding and night waking may also be related to breastfeeding mothers’ high sensitivity, leading to increased likelihood of responding to their infants’ signal, and in this case responding to each night waking with night breastfeeding (Britton et al., 2006).

In light of the possibilities above, frequent night waking as well as frequent night breastfeeding are a common and realistic practice among breastfeeding mother-infant pairs, and both, undeniably, are important factors in continuing breastfeeding during this age. More importantly, this relationship will likely reduce with age (Mindell et al., 2012) and will not likely to contribute to later sleeping problems (Hysing et al., 2014). However, the extent to which nocturnal awakenings constitute a sleep problem is currently unknown because the empirical basis for defining what should be regarded as normative sleep in early childhood is very limited.

In relation to sleep location, our data showed that half of the breastfed infants were bed-sharing with their mothers who were mainly ethnic Norwegian, which is line with national data (Osberg et al., 2021). As expected, we found that bed-sharing was associated with an increased likelihood of night waking. Similar findings were also shown in previous studies (Hysing et al., 2014; Mindell et al., 2012; Sadeh et al., 2009). These studies suggested that bed-sharing was an environmental factor that predicted increased night waking (Sadeh et al., 2009). Promoting of breastfeeding is one of the main reasons given for bed sharing, and getting more sleep is another leading reason (Bartick et al., 2022; Salm et al., 2015).

Breastfeeding has added benefits, as it has also been proven to reduce the likelihood of SIDS among infants (Moon, 2016). Despite this, bed-sharing is not recommended due to the increased risk of suffocation and SIDS (Moon, 2016). There are also different views on bed-sharing related to safe sleep and promoting breastfeeding. There are recommendations in Norway on bed sharing and related risky behaviors such as the consumption of tobacco or alcohol or unsafe sleeping places, as this increases cases of SIDS. There are several positive factors associated with breastfeeding and bedsharing, including increased sleep duration and a reduced risk of SIDS.

The recommendation for safe infant sleeping is for infants to sleep in their own crib in the same room as the parents, also known as room-sharing. The arrangement of room-sharing, however, is not as accessible as bed-sharing for breastfeeding and night waking, which could challenge breastfeeding continuity. Room-sharing, but not bed-sharing, could also pose a risk of parents falling asleep accidentally while holding their infants during night breastfeeding. Ultimately, the recommendations of safe sleeping for optimal breastfeeding practices and sleeping patterns needs further consideration.

Furthermore, our data showed that the breastfed infants slept for 11 h during the night. This was similar to previous studies’ findings which showed that nighttime sleep duration among infants at this age, regardless of breastfeeding status, was approximately 9–11 h per night (Hysing et al., 2014; Mindell et al., 2012; Sadeh et al., 2009).

In adjusted analysis, this study found that infants who were older (9–12 months) had longer daytime sleep duration, longer night wakefulness and were likely to have shorter nighttime sleep duration. However, although the difference was statistically significant, but we are not sure it’s clinically relevance. Infants who had not being vaccinated recently and who slept alone in their bed were likely to have a longer nighttime sleep duration. In line with previous findings from a large web-based study conducted by Sadeh et al. (2009), these present associations indicate that biological and ecological factors particularly room sharing, as well as sleep need, were related to the duration of night sleep.

Interestingly, our data showed that infants who were being frequently breastfed during the night were likely to sleep longer at night. It is possible that infants who were breastfed more frequently during the night only awake for a short duration and fall back to sleep more quickly than those who were breastfed less frequently during the night. A previous study on the potential role of melatonin content in breast milk by Engler et al. (2012) found decreased occurrence of both infantile colic and irritability, as well as longer nighttime sleep duration, among 2–4 months of infants who were exclusively breastfed (Engler et al., 2012). It was suggested that the high melatonin level secreted during the night may increase the nighttime sleep duration (Engler et al., 2012). Another study finding suggested that tryptophan, contained in night-breast milk, also had an effect in promoting nighttime sleep duration through melatonin synthesis (Cubero et al., 2005). Thus, it is possible that the more frequently infants are breastfed during the night, the higher the melatonin level among infants, which is associated with infants sleeping for a longer duration at night.

### Strengths and Limitations of the Study

The results from this study need to be interpreted carefully due to methodological limitations. As a partially web-based study, the respondents in this study were skewed towards highly educated mothers of Norwegian background. This was affirmed by the higher percentage of mothers with Norwegian backgrounds and university/college-level education in our present study compared to the national survey (Lande & Helleve, 2014). Consequently, the study's findings may be more representative of individuals sharing similar characteristics with our study population than of the entire Norwegian population. Moreover, we encountered challenges in recruiting participants through the Community Health Services, resulting in limited diversity in participants' socioeconomic status, educational attainment, and cultural backgrounds. Due to these recruitment limitations, consequently study results may not reflect the general Norwegian population.

Nonetheless, our use of the BISQ through both paper and online channels may have lowered the risk of research participation withdrawal due to its accessibility and practicality. The large number of participants who completed the study may have increased the sampling variability, and therefore the result derived may be closer to the population (Abramson & Abramson, 2008).

Sleep patterns were assessed using subjective measurements (parental reports), which can have an impact on the results due to level of awareness and recollection differences among the parents in relation to infants’ night waking. Nevertheless, BISQ is a valid measurement in assessing infant sleep patterns through the internet (Sadeh, 2004). Therefore, the results generated in the present study can also be compared to earlier internet-based studies (Sadeh et al., 2009).

Furthermore, a previous study has shown that the BISQ was significantly correlated with other objective and subjective measurements, including actigraphy and sleep diaries (Sadeh, 2004). Survey-derived nighttime sleep duration was suggested to be similar to the measures from actigraphy, while night waking frequency was less frequent than that measured by actigraphy but more accurate than the measure from sleep diaries (Sadeh, 2004). Therefore, while we used subjective measurements, the nighttime sleep duration is likely similar to the actual duration. Night waking frequency may be slightly less than the actual frequency. In future studies, it is recommended to use several methods of sleep measurement to increase internal validity. There is inconsistent evidence of the interplay between night waking frequency, night sleep duration, and breastfeeding among small children (Perrella et al., 2022). However, our findings add to the limited body of evidence on breastfeeding and frequent infant night waking.

## Conclusion

Our findings suggest that frequent breastfeeding and bed-sharing during the night among 6–12 month-old infants were related to a greater likelihood of frequent night waking. While infants who were frequently breastfed during the night were more likely to have a longer nighttime sleep duration. These findings hold the potential to significantly inform breastfeeding practices of parents and guide future research related to breastfeeding practices, bed sharing, and infants’ feeding practices during the first years of infancy. However, more in-depth research in this area is needed.

### Supplementary Information

Below is the link to the electronic supplementary material.

Supplementary file1 (DOCX 208 kb)
