# Supplementary material for: Breastfeeding and Sleeping Patterns Among 6–12-Month-Old Infants in Norway
Source: Matern Child Health J. 2023 Nov 19;28(3):496–505. doi: 10.1007/s10995-023-03805-2 (PMC10914878; doi:10.1007/s10995-023-03805-2)
Supplement: Supplementary file 1 — Supplementary file1 (DOCX 208 kb) [file 10995_2023_3805_MOESM1_ESM.docx]

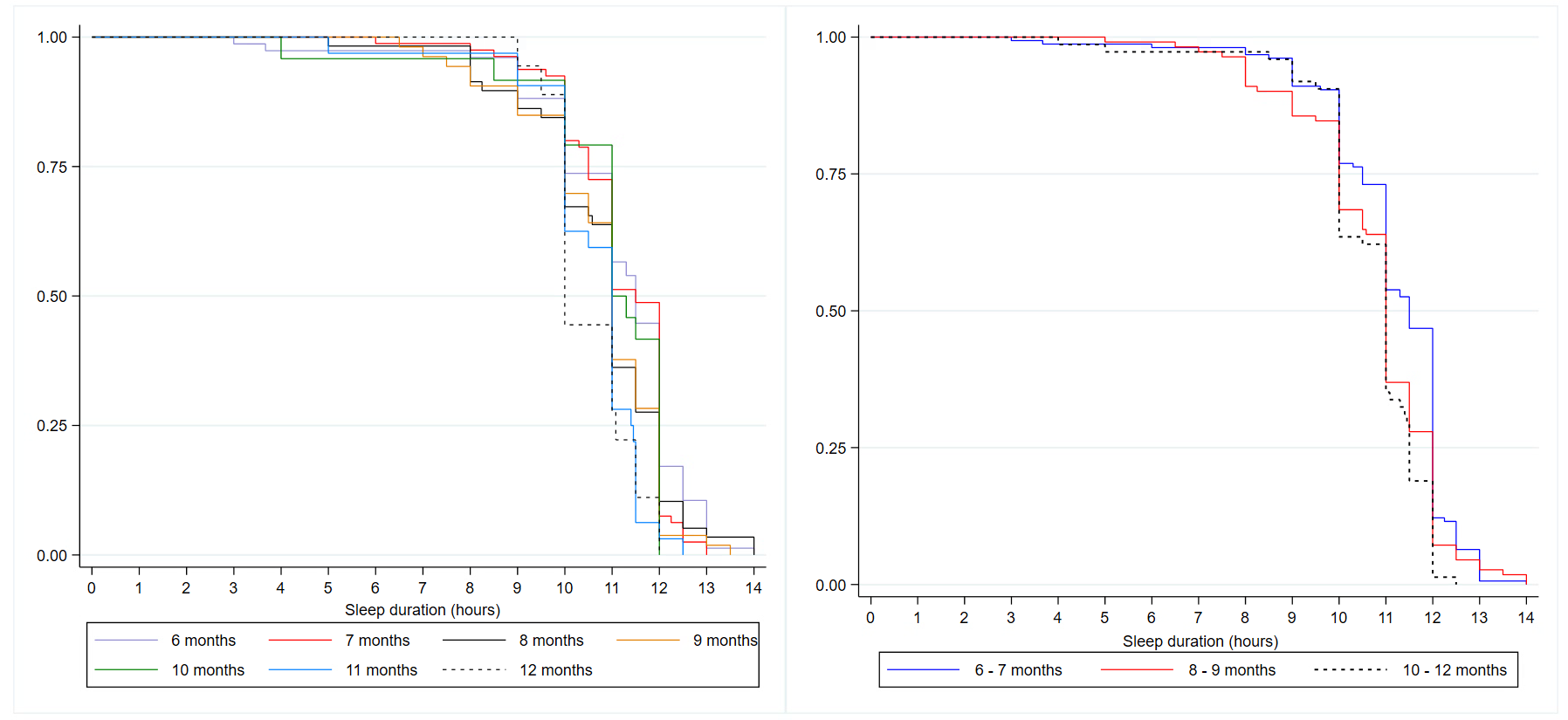


**Fig S1** Kaplan-Meier plot showing nighttime sleep duration of infants in all age groups (left panel) and three age groups 8right panel). A drop in the curve indicates the time infants woke up (that is, the end of the nighttime sleep duration)


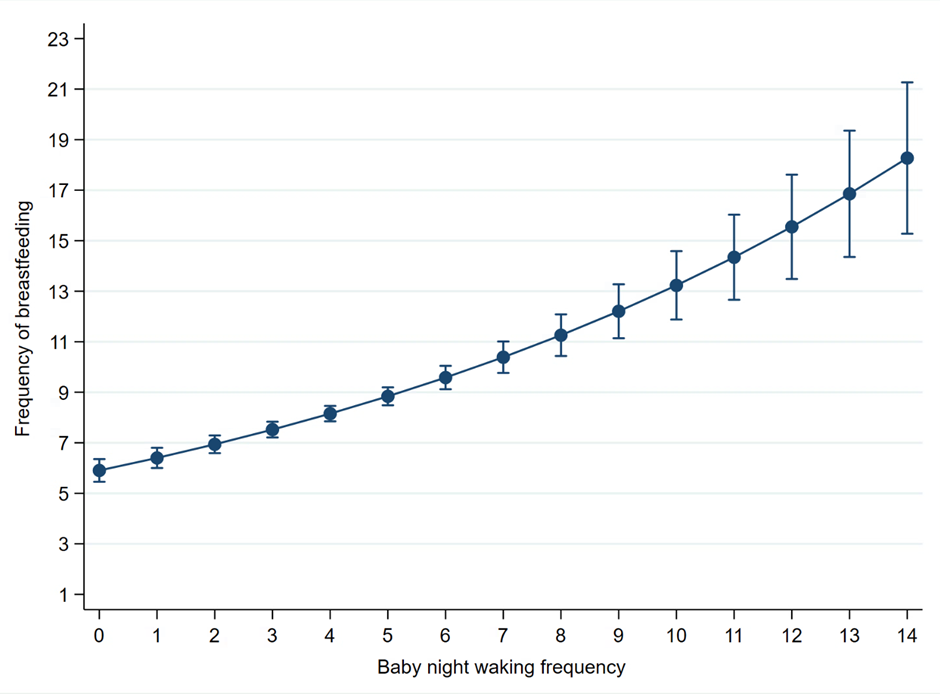


**Figure 2 S.** Marginsplot showing the association between baby night-waking frequency and night breastfeeding frequency
